# An Exploratory Study on the Impact of MIPEF-Assisted Extraction on Recovery of Proteins, Pigments, and Polyphenols from Sub-Standard Pea Waste

**DOI:** 10.3390/foods15010128

**Published:** 2026-01-01

**Authors:** Stella Plazzotta, Alberto Saitta, Sofia Melchior, Lara Manzocco

**Affiliations:** 1 Department of Agricultural, Food, Environmental and Animal Sciences, University of Udine, Via Sondrio 2/A, 33100 Udine, Italy; stella.plazzotta@uniud.it (S.P.); lara.manzocco@uniud.it (L.M.); 2 Department of Human Sciences and Promotion of the Quality of Life, San Raffaele University, Via Val Cannuta 247, 00166 Rome, Italy; sofia.melchior@uniroma5.it

**Keywords:** pulsed electric fields, electroporation, bioactive compounds, upcycling, legume by-products

## Abstract

The growing demand for sustainable protein sources has intensified the need for efficient valorisation of legume by-products. This study investigated the application of moderate intensity pulsed electric fields (MIPEF; 5 kV/cm, 4 μs, 500 pulses) as a green technology for assisting the co-extraction of proteins, pigments, and polyphenols from industrial substandard peas (*Pisum sativum* L.). Aqueous pea dispersions (20 g/100 g) were subjected to alkalinization (pH 9–12), and MIPEF applied either before or after the pH adjustment. The highest protein recovery was achieved when MIPEF was applied after alkalinization at pH 9.0, due to the increased conductivity and energy input enhancing electroporation-driven protein release. Although higher pH levels increased energy delivery, they did not significantly improve protein extraction. Conversely, MIPEF application decreased total polyphenol and pigment concentrations in the extract, likely due to aggregation phenomena. Overall, these preliminary results indicate that combining mild alkalinization with MIPEF might represent a promising and energy-efficient approach for protein recovery from legume side-streams. Further optimization is required to improve protein recovery while preserving the stability of co-extracted bioactive compounds.

## 1. Introduction

According to the FAO [1], the world population is expected to reach 9.7 billion by 2050, resulting in a significant increase in food demand and placing considerable pressure on agricultural and food industrial systems. The United Nations 2030 Agenda for Sustainable Development emphasizes the need to guarantee food security, ensuring equitable food distribution while safeguarding the planet’s health by eradicating hunger and promoting sustainable production and consumption models (Sustainable Development Goals 2 and 12) [2]. However, achieving these goals implies addressing multiple challenges, including climate change, soil degradation, biodiversity loss, and inefficiencies across the food supply chain. In this context, animal food production is deemed responsible for the unsustainable exploitation of resources such as land and water, while producing large amounts of greenhouse gases [3]. For this reason, increasing importance is attributed to the so-called “protein transition”, which refers to the increasing replacement of animal proteins with more sustainable alternatives such as legumes, algae, or insects. Legumes are particularly promising, since they are: (i) already widely known by consumers; (ii) rich in proteins, fibres and in bioactive compounds, such as polyphenols; (iii) well recognized for their antioxidant, antimicrobial, and anti-inflammatory effects [4]. The demand for legumes has been thus experiencing a constant increase in recent years [5]. However, this growing demand has also increased the amount of processing waste generated along legume supply chains. In particular, a remarkable fraction of legume production is discarded as substandard materials, referring to seeds showing irregular shape, colour, or size, commonly removed at the entrance of industrial processing lines by automatic optical selectors. Although substandard seeds are nutritionally equivalent to marketable legumes, they are typically downcycled to the production of animal feed or biogas [6]. However, when these waste management options are adopted, the high-value proteins, pigments and polyphenols of substandard legumes are not fully exploited. In particular, based on the estimation of a large Italian company, substandard seeds from peas (*Pisum sativum* L.) represent up to 15% of processed peas, accounting for 0.135 million tons/year of waste in the EU only. Substandard peas are rich in proteins (20–25% of dry weight [7,8]), increasingly requested by consumers for their nutritional and functional properties. The latter, combined with low allergenicity, make peas an attractive alternative to soy [9,10,11]. Nevertheless, the available literature on pea waste valorisation via protein extraction has primarily concentrated on pod residues [12,13,14], whilst studies investigating protein extraction from peas typically employ edible peas or commercial pea flours [15,16,17].

At the same time, pea polyphenols have shown antioxidant, antibacterial, and anti-inflammatory activities [18], which have attracted extensive attention from many researchers [19]. Pea polyphenols include flavonoids (apigenin, quercetin and kaempferol), phenolic acids (mainly derivatives of hydroxybenzoic and hydroxycinnamic acid) and tannins (mainly catechins) [20]. Peas are also rich in pigments, mainly chlorophylls and carotenoids, which confer the typical bright green colour [21].

The recovery of proteins, polyphenols and pigments from plant matrices is traditionally achieved through solvent-based extraction combined with mechanical shear, aiming at disrupting the vegetable tissue and favouring biomolecule release from the fibrous plant structure they are strongly embedded in. Although widely used, these methods generally involve high energy consumption, increased risk of target compound degradation and low yields [22]. To overcome these limitations, solvent extraction methods can be optimized to avoid organic solvent use, preserve solute integrity and/or increase the recovery yield. Some effective examples include supercritical and subcritical extraction, microwave- or ultrasound-assisted extraction and solid phase extraction [23,24]. In this context, pulsed electric field (PEF) technology has emerged as a low-energy technology able to assist extraction to increase recovery yield while often using water as the main solvent. PEF operates by applying short, high-voltage pulses (micro- to milliseconds) that induce a transmembrane potential difference, causing electroporation, i.e., the formation of pores across cell membranes [25]. Two intensity ranges are typically distinguished: high-intensity PEF (HIPEF), with electric field strengths exceeding 20 kV/cm, mainly used for microbial inactivation during non-thermal pasteurization [26]; and medium-intensity PEF (MIPEF), which applies electric fields usually in the range from 0.2 up to 10 kV/cm, mainly employed to assist extraction. Indeed, this process enhances permeability and facilitates the release of intracellular compounds, such as soluble proteins, polyphenols and pigments.

Recent studies have highlighted the potential of MIPEF in legume processing [27,28]. For instance, Baier et al. [16] reported that MIPEF treatment of dry peas at 5 kV/cm significantly improved hydration kinetics, reducing soaking and precooking time. Some studies also exploited MIPEF in the extraction of target compounds from legume by-products: for instance, Guo et al. [29] demonstrated that MIPEF can be used to enhance the extraction of polyphenols from pea hulls, resulting in bioactive extracts with hepatoprotective properties. Similarly, in the study of Morales-de la Peña et al. [30], MIPEF application to soy slurry allowed increasing the concentration of total phenolic compounds, betalains, and isoflavones in the derived plant-based beverage. Even if no specific studies have been performed on legume industry by-products, MIPEF treatment has also been shown to improve the extraction of soluble proteins from herbs and algae [31,32]. Additionally, MIPEF has been used to favour the extraction of pigments, such as chlorophylls and carotenoids, from discarded vegetable matrices [33,34,35]. However, some authors have reported that PEF may also lead to the loss of pigments and other bioactive compounds, especially when applied to industry-generated vegetable by-products. Differently from waste material freshly prepared in the laboratory, industrial waste material is commonly stabilized via freezing or drying, treatments that are known to strongly damage cells, promoting the release of target compounds, making them more sensitive to subsequent treatment [36]. The final effect of PEF treatments is hence hardly predictable, especially when targeting the co-extraction of multiple compound classes from industrial waste [37,38].

Based on these considerations, the aim of this study was to collect preliminary data to evaluate the impact of MIPEF as an assisting technology for the extraction of proteins from industrially generated substandard peas, while also considering co-extracted compounds such as polyphenols and pigments. To this aim, following the identification of the treatment able to maximise protein recovery, such conditions were tested in terms of polyphenol and pigment extraction.

## 2. Materials and Methods

### 2.1. Raw Vegetable Material

Substandard peas were collected at Conserve Italia Soc. Coop. Agricola (Pomposa, Italy), manually cleaned to remove any pods, leaves, or insects, and subjected within 6 h to a blanching process by immersion in water at 90 °C for 5 min, followed by blast freezing (AOFPS061, Electrolux, Pordenone, Italy) at −30 °C for 45 min and storage at −18 °C in a freezer (REX71FF, Electrolux, Pordenone, Italy) until use.

### 2.2. Preparation of Substandard Pea Dispersions

**Figure 1:**
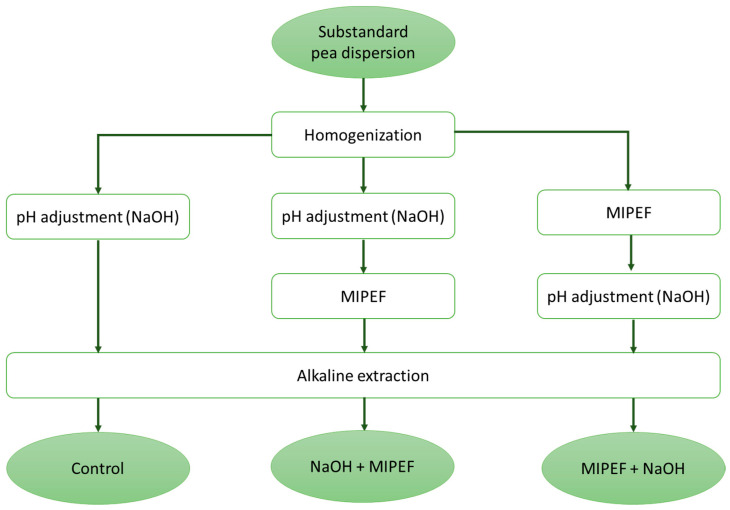
Experimental plan followed for the preparation of substandard pea extracts by applying pH adjustment, MIPEF followed by pH adjustment and pH adjustment followed by MIPEF.

Before extraction, known quantities of frozen peas were thawed in a refrigerator overnight at 4 °C (REX71FR, Electrolux, Pordenone, Italy). Thawed peas (6 g) were then supplemented with double-distilled water (System Advantage A10^®^, Millipore S.A.S., Molsheim, France) (24 g) to obtain a 20% *w*/*w* dispersion and finely homogenized using a high-speed homogenizer (Ultra-Turrax T18, IKA^®^-Werke GmbH & Co. KG, Staufen im Breisgau, Germany) for 10 min at 10,000 rpm. Homogenization was carried out at room temperature. During homogenization, temperature was measured with a copper-constantan thermocouple probe (Ellab, Hillerød, Denmark) connected to a portable data logger (mod. 502A1, Tersid, Milan, Italy). The dispersions were then subjected to (i) pH adjustment; (ii) pH adjustment followed by MIPEF; (iii) MIPEF followed by pH adjustment. The details relevant to pH adjustment and MIPEF are reported in Section 2.3 and Section 2.4, respectively. The extracts were then subjected to alkaline extraction (see Section 2.5). The obtained extracts were respectively labelled as (i) Control; (ii) NaOH + MIPEF; (iii) MIPEF + NaOH. Figure 1 shows a scheme of the experimental plan followed and the obtained samples.

### 2.3. pH Adjustment

The pea dispersions were supplemented with 6 M sodium hydroxide (NaOH, Sigma-Aldrich, Milan, Italy) under magnetic stirring (Microstirrer, VELP© Scientifica Srl, Usmate, Italy) at room temperature until reaching a pH of 9, 10, 11, or 12 (Hanna Instruments pH 301, Padua, Italy). The time needed to adjust the pH was between 5 and 6 min, depending on the final pH setpoint.

### 2.4. MIPEF Treatment

**Figure 2:**
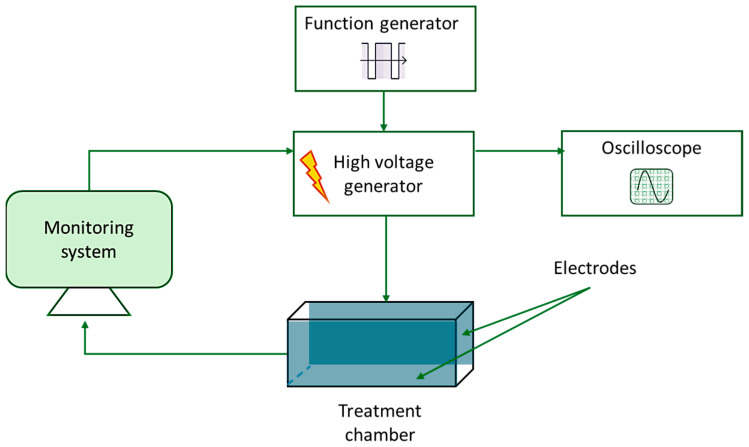
Scheme of the MIPEF plant used for the experimentation.

For the medium-intensity pulsed electric field (MIPEF) treatments, the lab-scale apparatus installed at the Department of AgriFood, Environmental and Animal Sciences of the University of Udine and schematized in Figure 2 was used. The system consisted of a treatment chamber connected to a high-voltage pulse generator (ScandiNova, System AB, M100, Uppsala, Sweden). The characteristics of the electrical pulses were controlled by a function generator (Tektronix AFG3022C, Tektronix Inc., Beaverton, OR, USA) and monitored using an oscilloscope (Tektronix MDO3034, Tektronix Inc., Beaverton, OR, USA). The generator was operated through the ScandiNova software (ScandiNova System AB, version 0.79, Uppsala, Sweden), while the treatment parameters were set using the LabVIEW4PEF software (LabVIEW4PEF_B-618-01 9.0, ProdAl, Fisciano, Italy). The treatment chamber (4.5 × 2.5 cm, with a gap of 1.5 cm) was filled with 15 × 10^−3^ kg (*m*) of pea dispersions, ensuring complete filling of the chamber. After loading, the outer surfaces of the cell were carefully inspected and cleaned in case of any sample leakage. Based on preliminary trials aiming at developing an electric field of about 5 kV/cm, the treatments were carried out by applying an input voltage (*U_0_*) of 350 V, a pulse frequency of 300 Hz, a pulse duration (*τ*) of 4 μs (4 × 10^−6^ s) and a number of pulses (*n*) of 500. After the treatment, the samples were centrifuged at 15,000× *g* for 5 min at 4 °C using a microcentrifuge (D3024, LABBOX Italia s.r.l., Milan, Italy). The resulting supernatant was collected by percolation and frozen until subsequent analysis. The supernatant was thawed at 4 °C overnight prior to analysis.

The monitoring system allows recording the following MIPEF output parameters: peak voltage in the chamber (*U_k_*, kV), transformation ratio (Rf, calculated as the ratio between the output *U_k_* and input *U_0_* voltage), current intensity measured at the treatment chamber (*I_k_*, A), electrical resistance (*R*, Ω) of the sample, and electrical conductivity (*σ*, mS/cm).

The software also calculates the electrical field (*E*, kV/cm). Specific energy per pulse (*W_p_* (kJ/(kg pulse))) and total energy (*W_t_*, kJ/kg) were determined according to the following equations (Equations (1)–(3)):

(1) $$E\;\;\left(\frac{kV}{cm}\right)=\frac{U_{k}}{gap}$$

(2) $$W_{p}\;\;\left(\frac{kJ}{kg\;pulse}\right)=\frac{U_{k}^{2}\;\tau }{R\;m\;1000}$$

(3) $$W_{t}\;\;\left(\frac{kJ}{kg}\right)=W_{p}n$$

### 2.5. Alkaline Extraction

Selected pea waste dispersions, i.e., those simply adjusted to pH 9.0 or adjusted to pH 9.0 and preceded or followed by MIPEF, were maintained under gentle stirring for increasing durations (15, 30, 45 and 60 min) (Figure 1).

### 2.6. Extract Separation

Multiple aliquots (2 mL) of the dispersions obtained after the different treatments were centrifuged using a microcentrifuge (Mikro 120, Hettich Zentrifugen, Andreas Hettich GmbH and Co., Tuttlingen, Germany) at 15,000× *g* for 5 min at 4 °C. The obtained supernatant (i.e., the extract) was collected and stored in the dark at −18 °C until analysis. Prior to analysis, the supernatant was thawed at 4 °C.

### 2.7. Dry Matter

The dry matter of substandard peas was determined gravimetrically according to AOAC [39]. To this aim, approximately 10 g of seeds was dried in a vacuum oven (1.32 kPa) at 75 °C to a constant weight (12 h).

### 2.8. Optical Microscopy

Samples were observed at room temperature using a Leica DM 2000 optical microscope, equipped with polarization lenses (Leica Microsystems, Heerburg, Switzerland). The images were taken at 40×, 100×, 200× and 400× magnification using a Leica EC3 digital camera (Leica Microsystems, Heerbrugg, Switzerland), and elaborated with the Leica Suite Las EZ software version 3.4 (Leica Microsystems, Heerburg, Switzerland).

### 2.9. Particle Size Distribution

The particle size distribution of the ground pea dispersion was determined using a MasterSizer 3000+ laser analyser (Malvern Instruments, Malvern, Worcestershire, UK). The analysis was carried out using water as the dispersant, with a laser obscuration rate of 10% and a stirring speed of 2500 rpm. The refractive indices set for the sample and for water were 1.33 and 1.58, respectively.

### 2.10. Protein Content

The quantification of total proteins in substandard peas was determined with the Kjeldahl method [40] by applying a nitrogen-to-protein conversion factor of 5.40, commonly applied for legumes [41,42]. For the quantification of the protein in the extracts, the bicinchoninic acid (BCA) method was applied using a microplate reader (Sunrise-basic Tecan, Tecan Austria, Grödig, Austria). The working reagent (WR) was prepared by mixing Reagent A, consisting of a bicinchoninic acid solution (BCA solution, Sigma-Aldrich, Milan, Italy), with Reagent B, a 4 g/100 mL copper(II) sulphate solution (Sigma-Aldrich, Milan, Italy), in a 50:1 (*v*/*v*) ratio. In each plate well, 25 μL of extract and 200 μL of WR were added. The plate was covered, placed in the reader, and shaken for 30 s, followed by incubation at 37 °C for 30 min. The absorbance was then measured at 562 nm. A blank was prepared by replacing the extract with double-distilled water. Protein concentrations were determined by comparison with a standard calibration curve prepared using bovine serum albumin (BSA, Sigma-Aldrich, Milan, Italy) in the 25–2000 μg/mL range (R^2^ = 0.9901).

Protein extraction yield was calculated as the percentage ratio between the amount of extracted proteins and the total protein content in the substandard peas subjected to extraction.

### 2.11. Polyphenol Content

The determination of the total polyphenol content (TPC) in the extracts was determined by using the Folin–Ciocalteu method [43]. An aliquot of 250 μL of supernatant, 12.5 mL of Milli-Q water, and 1.25 mL of Folin–Ciocalteu reagent (Carlo Erba, Milan, Italy) were mixed in a volumetric flask. After 30 s, 5 mL of 15 g/100 mL sodium carbonate solution (Na_2_CO_3_, Carlo Erba, Milan, Italy) was added, and the volume was made up to 25 mL with Milli-Q water. The mixture was incubated in the dark for 2 h, and the absorbance was then measured at 750 nm (UV-2501 PC, Shimadzu, Kyoto, Japan) against a blank prepared by substituting the supernatant with distilled water. Results were expressed as mg of gallic acid equivalents (GAE) per g of substandard pea dry matter, calculated from a standard calibration curve (R^2^ = 0.9974) prepared using gallic acid (Sigma-Aldrich, Milan, Italy) as reference standard.

### 2.12. Pigment Content

The methodology of Alotaiby et al. [44] was followed. The extracts were analysed using a UV–Vis spectrophotometer (Shimadzu UV-2501PC, Shimadzu Corporation, Kyoto, Japan) at the following wavelengths: 663, 645 and 470 nm. The concentration of chlorophylls and total carotenoids (μg/mL) was determined by the following equations (Equations (4)–(6)):

(4) $$C_{chlorophyll\;a}\;\left(\frac{\mu g}{mL}\right)=\;12.25\;\times \;A_{663}\;-\;2.79\;\times \;A_{645}$$

(5) $$C_{chlorophyll\;b}\;\left(\frac{\mu g}{mL}\right)=21.50\times A_{645}-5.10\times A_{663}$$

(6) $$C_{carotenoids}\;\left(\frac{\mu g}{mL}\right)=\frac{1000\times A_{470}-1.82C_{chlorophyll\;a}-85.2C_{chlorophyll\;b}}{198}$$

where A is absorbance (a.u.) at the corresponding wavelength for the extract. The concentration of each pigment was expressed as a fraction of the dry matter of substandard peas (μg/g).

### 2.13. Data Analysis

The reported data are expressed as the mean and standard deviation of at least two measurements from at least three replicates (*n* = 3). The statistical analysis was carried out using the program R version 4.2.2 (The R Foundation for Statistical Computing, Wien, Austria). The normality of the data distribution was assessed using the Shapiro–Wilk test, and homogeneity of variance was evaluated with the Bartlett test. Differences between group means were analysed using a one-way or two-way ANOVA, as appropriate, followed by Tukey’s post hoc test (*p* < 0.05). For comparisons between two groups, a paired sample *t*-test was conducted with significance set at *p* < 0.05.

## 3. Results and Discussion

**Figure 3:**
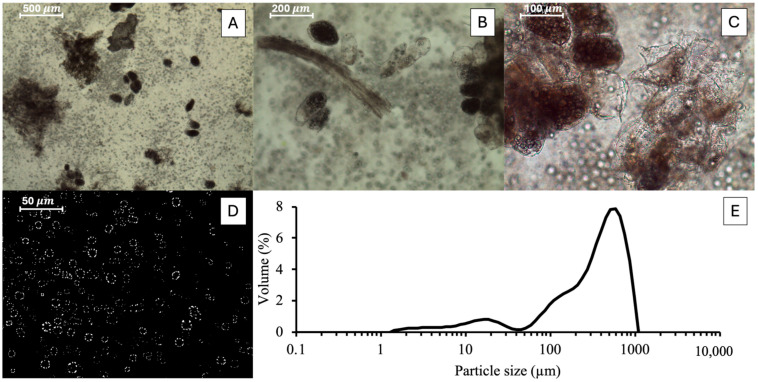
Microscopic image of ground substandard pea dispersion at magnification of 40× (**A**), 100× (**B**), 200× (**C**) and 400× (**D**), and relevant particle size distribution (**E**).

In the first part of the study, attention was focused on the identification of the process conditions allowing for maximising protein extraction. Proteins are indeed the main target when dealing with substandard peas, due to the rich protein content of this by-product [4]. In particular, the used substandard peas presented a dry matter of 22 g/100 g fresh weight and a protein content of 5.43 g/100 g fresh weight, in line with the composition of standard peas [45,46]. In this regard, it should be noted that substandard peas are discarded mainly due to aesthetic defects, including out-of-size dimension, irregular shape and yellowish surface colour. Since efficient protein extraction from legumes requires extensive cell disruption [47,48], substandard peas were subjected to fine grinding to maximise intracellular protein availability. In this regard, Figure 3A–D reports the micrograph of the pea dispersion upon grinding at increasing magnification, while the relevant particle size distribution is shown in Figure 3E.

The microscopic image taken at the lowest magnification (Figure 3A) showed the presence of large particles with dimensions of several hundreds of μm, represented by tissue fragments with intact cells. At larger magnification (Figure 3B), rod-like particles, attributable to fibrous fragments, with a radial dimension of around 50 μm were identified, along with small clusters of vegetable cells. At 200× (Figure 3C), granule structures both within intact cells and in the surrounding medium were identified. These structures are attributable to starch granules, presenting dimensions around 10–20 μm, recognizable from their characteristic cross-shaped birefringence (Maltese cross), clearly visible at the highest magnification obtained in polarized light mode (Figure 3D). The presence of particles with heterogeneous dimensions was confirmed by particle size analysis (Figure 3E). The distribution shows a first, broad signal in the 5–50 μm range, which correlates with the presence of small cellular clusters and granular structures (Figure 3B,C). A second, dominant peak appeared between 300 and 1000 μm, attributable to the large particles observed in Figure 3A.

The obtained dispersions, showing a native pH of 6.7 ± 0.2, were then subjected to pH adjustment to pH 9.0. Proteins are indeed well-known to be efficaciously extracted at alkaline pH since, under these conditions, the ionization of protein aminoacidic residues occurs, associated with the breakage of disulfide cross-linking and fibre solubilization. In this way, proteins are effectively released from the fibrous matrix [49].

The adjustment of pH was associated with significant changes in the electrical resistance and conductivity of the substandard pea dispersions, as shown in Table 1.

In particular, the electrical resistance of the dispersion adjusted to pH 9.0 was significantly lower, while its conductivity was significantly higher compared with the dispersion at native pH. This is consistent with the higher electrolyte concentration in the alkalinized solution, resulting from NaOH dissociation in the aqueous medium and the release of solutes from the matrix through alkaline hydrolysis [50]. Such changes are expected to affect the MIPEF treatments, since they directly influence the distribution and intensity of the electric field within the sample, thereby possibly altering the overall effectiveness of the treatment. The substandard pea dispersions with native pH (6.7) or pH 9.0 were thus subjected to MIPEF treatments, and output parameters were recorded (Table 1). In both cases, the measured voltage (*U_k_*) in the treatment cell was around 8 kV, accounting for a transformation ratio (*R_trasf_*) slightly higher than 23, which was due to the presence of a voltage step-up system in the pulse generator, amplifying the nominal input voltage (350 V). In agreement with the similarly developed voltage, the electric field strength within the treatment chamber, calculated as the ratio between the voltage and the gap between the electrodes, was consistently around 5 kV/cm.

By contrast, both current and specific energies significantly changed according to the pH of the treated dispersion. In particular, the current (*I_k_*) measured in the sample subjected to alkalinization at pH 9.0 was about 25% higher than that recorded for the sample treated at its native pH, which should be attributed to the higher conductivity and lower electrical resistance detected in this dispersion (Table 1), as previously observed for PEF treatments of plant matrices with different electrical conductivities [51]. Accordingly, the specific pulse (*W_p_*) and total energy (*W_t_*) delivered during the MIPEF treatment of the alkalinized sample was 22% higher than that observed for the non-alkalinized dispersion. It is reasonable to assume that these differences in MIPEF treatment intensity could affect protein extraction yield.

**Figure 4:**
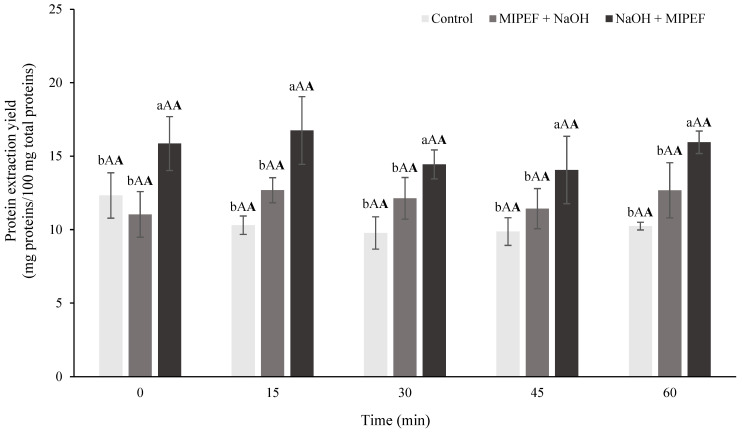
Protein extraction yield determined after extraction for increasing time duration of substandard pea dispersions subjected to alkaline extraction at pH 9.0 (Control), MIPEF at native pH followed by alkalinization at pH 9.0 (MIPEF + NaOH), adjustment to pH 9.0 followed by MIPEF treatments (NaOH + MIPEF). Different lower-case letters (a, b) indicate significant differences among treatments. Different upper-case letters (A) indicate significant differences among sampling times. Different bold upper-case letters (**A**) indicate significant treatment × time interaction effects. Statistical analysis was performed using two-way ANOVA followed by Tukey’s post hoc test (*p* < 0.05).

To verify this hypothesis, the dispersions were subjected to alkaline extraction at pH 9.0 for up to 60 min. To this aim, the MIPEF-treated dispersions at native pH were adjusted to pH 9.0 (MIPEF + NaOH), while those adjusted to pH 9.0 before MIPEF treatments (NaOH + MIPEF) were used without further modification (Figure 1). A control sample was also prepared, consisting of a substandard pea dispersion adjusted to pH 9.0 and not subjected to MIPEF treatment. In this regard, it must be underlined how an alkaline medium is well-known to promote protein solubilization, driven by ionization of charged amino acid residues, and is thus widely applied for protein extraction [52]. The protein extraction yield measured in these samples is shown as a function of extraction time in Figure 4.

Unexpectedly, as shown in Figure 4, extraction time was proven to be an uninfluential variable in determining protein extraction yield. Indeed, for each treatment, the protein yield remained relatively unchanged with the increase in extraction time, suggesting that protein release was already efficiently performed in the steps preceding the alkaline extraction step. This can be attributed to the sample preparation procedure, which involved intensive grinding of a relatively small amount (20 g) of substandard pea dispersions for 10 min. Such vigorous homogenization caused a substantial disruption of plant tissue, promoting cell rupture into small particles and subsequent protein release (Figure 3) [53]. It must also be noted that alkaline extraction procedures applied to recover proteins from different vegetable sources show that most proteins are released in the first 10–15 min [17,54], suggesting that the applied grinding step provided adequate time to approach the extraction plateau. Also, during grinding, the temperature increased by approximately 15 °C, reaching around 35 °C, which may have further facilitated protein solubilization [55]. Furthermore, results reported in Figure 4 show that the application of MIPEF before pH adjustment (MIPEF + NaOH) did not promote a significant increase in protein extraction yield as compared to the Control. This suggests that, without the solubilizing effect of alkaline conditions, MIPEF alone was insufficient to promote a measurable increase in protein extraction from ground pea matrices. By contrast, a significantly higher extraction yield was observed when the ground pea dispersion was subjected to MIPEF after pH adjustment (NaOH + MIPEF). This is probably related to the higher energy input of this treatment, as shown in Table 1, which is known to enhance PEF-induced effects [51]. In particular, MIPEF likely increased the release of proteins from the remaining intact cellular structures (Figure 3A) into the surrounding alkaline medium, due to electroporation phenomena, which intensify with increasing energy delivery [27]. Additionally, MIPEF application may have promoted physicochemical changes in the proteins or in their surrounding matrix, favouring the disruption of bonds between proteins and non-protein compounds (such as fibres, starch or polyphenols), which often hinder protein extraction [56]. In support of this hypothesis, it has been demonstrated that MIPEF treatments are able to significantly alter the structure of proteins, by inducing unfolding and intramolecular rearrangement [57]. In some systems (e.g., gluten solution at pH 5.0, soybean slurry), this was shown to increase protein solubility, due to the ability of electric treatments to promote unfolding of insoluble aggregates [30].

Based on the obtained results, protein extraction yield can be increased by acting on the conductivity of the pea protein dispersion, which is normally performed during a standard alkaline extraction protocol by increasing the pH. To further confirm this effect, the MIPEF treatments were applied to substandard pea dispersions adjusted to pH 10.0, 11.0, and 12.0, which are in the range of those commonly applied for protein extraction [52]. Table 2 reports the electrical conductivity and resistance of the dispersions, as well as the output treatment parameters.

As expected, the increase in dispersion pH from 9.0 (Table 1) to 12.0 led to a progressive increase in electrical conductivity (*σ*), accompanied by a decrease in resistance (*R*), which accounted for a progressively higher current intensity (*I*) and specific pulse and total energy (*W_p_* and *W_t_*). These results can be explained by the greater availability of negative ions at higher pH values, due to both the higher NaOH concentration and the deprotonation of –COOH groups in proteins and polysaccharides under progressively stronger alkaline conditions. This phenomenon, in turn, promotes a higher charge flow, leading to an increase in electrical conductivity in the treated sample [58,59]. Nevertheless, protein extraction yield observed upon MIPEF treatment of dispersions adjusted to pH 10.0, 11.0 and 12.0 did not increase significantly as compared to the one observed when MIPEF treatments were applied to dispersions adjusted to pH 9.0 (Table 1). It must also be noted that the obtained extraction yields did not differ significantly from those registered under alkaline extraction conducted at pH 10.0, 11.0 and 12.0 without MIPEF application. These results indicate that under the applied conditions, pH values higher than 9.0 are not effective in improving protein extraction, whether in association with MIPEF or not. It can be inferred that, given the limited number of intact cellular structures remaining after grinding (Figure 3A–C), most of the extractable proteins were already solubilized at pH 9, so further increasing alkalinity did not provide additional benefits to protein recovery. Moreover, alkaline pH might also facilitate the co-extraction of non-protein components, such as polyphenols and pigments, which could interact with proteins and decrease the amount of extractable protein. To verify this hypothesis, additional experiments were carried out by assessing the content of phenolic compounds and pigments in the pea extracts exposed to MIPEF treatments. The sample adjusted to pH 9.0 followed by MIPEF treatment (NaOH + MIPEF, Figure 1) was selected for further investigation, as it resulted in the highest protein recovery (Figure 4). The extract obtained without MIPEF application was considered as control (Figure 1). Table 3 reports the obtained results.

The results reported in Table 3 show that the application of MIPEF at pH 9.0 led to extracts with a lower concentration of both polyphenols and pigments. In particular, the TPC and the content of chlorophyll A, B and carotenoids detected in the MIPEF-derived extract were 85, 61, 70 and 61% lower, respectively, than those of the corresponding control extract (Table 3). A similar decrease in biocompounds was also observed in our previous paper relevant to MIPEF-assisted extraction from industry-generated peach pomace stabilized through freezing [60]. A reduction in specific compounds following PEF treatment has been previously reported. For instance, a decrease in anthocyanins in tomato juice and vitamin C in model systems was observed upon PEF treatment [37,38]. However, it must be acknowledged that a large body of literature reports PEF as an effective technique to assist the extraction of polyphenols, chlorophylls, and carotenoids from different vegetable materials such as spent grain and algae [61,62,63,64]. Therefore, the reduced recovery observed in this study (Table 3) likely does not arise from PEF-driven degradation of pigments and polyphenols. Rather, it could be inferred that PEF treatment might trigger interactions among released compounds, limiting their solubility and extraction. In this regard, although little information is available on the underlying mechanisms of PEF-induced interactions among cellular components, recent evidence suggests that PEF treatments modify the organization of grape skin cell walls, indicating interaction between fibres and polyphenols, with variable effects on polyphenol concentration in the final extracts [65]. The fibre release observed upon grinding (Figure 3A) could be expected to favour these interactions. Similarly, polyphenols are known to be highly reactive to the formation of complexes with starch [66], which was effectively released by the applied grinding operations, potentially favouring these interactions (Figure 3A). Likewise, the strong affinity of polyphenolic compounds for protein side chains favours protein–polyphenol interaction through multiple binding mechanisms, involving hydrogen bonding, hydrophobic interactions, and occasional covalent linkages [20]. Regarding pigments such as chlorophyll molecules, they are known to spontaneously form chlorophyll aggregates in solution due to intermolecular interactions such as hydrophobic bonding and π–π stacking [67].

## 4. Conclusions

The present study demonstrated that medium-intensity pulsed electric fields (MIPEF) can enhance alkaline-assisted protein extraction from industrial substandard peas under specific alkaline conditions. Indeed, the highest protein recovery was achieved when MIPEF was applied after alkalinization at pH 9.0, owing to the increased conductivity and energy input that intensified electroporation phenomena. Increasing the pH above 9.0 up to 12.0 led to a further increase in treatment energy but did not improve extraction yields. From an industrial perspective, operating at pH 9.0 would present the advantage of minimizing the use of chemicals for both alkalinization and downstream neutralization, positively affecting process efficiency, cost, and sustainability.

Conversely, MIPEF application reduced the concentrations of polyphenols and pigments, possibly due to the aggregation of bioactive compounds released from the plant matrix. This approach highlights that the energy delivered by PEF could be synergistically combined with pH-driven protein solubilization. However, other PEF parameters (e.g., different electric field intensities, pulse widths, and specific energy inputs) may be tuned to further optimize the process, aiming at mitigating losses of non-protein co-extracted biocompounds. For example, assessing MIPEF-assisted extraction on pea waste subjected to milder grinding conditions could help preserve co-extracted bioactive compounds, while better isolating the intrinsic contribution of MIPEF to extraction. Future work will thus aim to optimize MIPEF extraction parameters to enhance pigment and polyphenol recovery, supported by targeted analytical techniques to elucidate the mechanisms governing their fate.

## Figures and Tables

**Table 1:** Electrical resistance (*R*) and conductivity (*σ*) of substandard pea dispersions at native pH or at adjusted pH 9.0. Peak voltage (*U_k_*), transformation ratio (*R_trasf_*), electrical field (*E*), current (*I_k_*), pulse specific energy (*W_p_*) and specific total energy (*W_t_*) of MIPEF treatments applied to the dispersions are also reported.

Dispersion pH	*R* (Ω)	*σ* (mS/cm)	*U_k_* (kV)	*R_trasf_* (−)	*E* (kV/cm)	*I_k_* (A)	*W_p_* (kJ/(kg pulse))	*W_t_* (kJ/kg)
6.7 (native)	254.1 ± 7.0 ^a^	0.52 ± 0.01 ^b^	8.27 ± 0.12 ^a^	23.62 ± 0.32 ^a^	5.51 ± 0.08 ^a^	32.55 ± 0.45 ^b^	2.36 ± 0.05 ^b^	1178 ± 23 ^b^
9.0	198.4 ± 4.8 ^b^	0.62 ± 0.13 ^a^	8.07 ± 0.09 ^b^	23.02 ± 0.28 ^a^	5.34 ± 0.00 ^b^	40.63 ± 1.11 ^a^	3.01 ± 0.09 ^a^	1506 ± 45 ^a^

a, b in the same column, means indicated by different letters are significantly different (*p* < 0.05).

**Table 2:** Electrical resistance (*R*) and conductivity (*σ*) of substandard pea dispersions adjusted to pH 10.0, 11.0 and 12.0. Current (*I_k_*), pulse specific energy (*W_p_*) and specific total energy (*W_t_*) of MIPEF treatments applied to the dispersions are also reported.

Dispersion pH	*R* (Ω)	*σ* (mS/cm)	*I_k_* (A)	*W_p_* (kJ/(kg pulse))	*W_t_* (kJ/kg)	Protein Extraction Yield (%)
10.0	151.0 ± 9.4 ^a^	0.89 ± 0.05 ^b^	53.13 ± 3.32 ^b^	3.81 ± 0.20 ^b^	1904 ± 99 ^a^	15.5 ± 1.1 ^a^
11.0	143.4 ± 4.3 ^a^	0.93 ± 0.03 ^b^	55.86 ± 1.65 ^b^	3.98 ± 0.09 ^b^	1993 ± 46 ^a^	14.6 ± 2.3 ^a^
12.0	103.8 ± 10.8 ^b^	1.30 ± 0.13 ^a^	76.56 ± 6.63 ^a^	5.16 ± 0.37 ^a^	2578 ± 183 ^b^	15.0 ± 1.9 ^a^

a, b in the same column, means indicated by different letters are significantly different (*p* < 0.05).

**Table 3:** Total phenolic content (TPC) and concentration of pigments (chlorophyll A and B, and carotenoids) in the extracts obtained upon alkalization at pH 9.0 of substandard pea dispersions followed (MIPEF) or not (Control) by MIPEF treatments.

Extract	TPC (mg_GAE_/g_dm_)	Chlorophyll A (μg/g_dm_)	Chlorophyll B (μg/g_dm_)	Carotenoids (μg/g_dm_)
Control	1.40 ± 0.01 ^a^	49.7 ± 4.9 ^a^	21.3 ± 1.2 ^a^	16.1 ± 0.3 ^a^
MIPEF	1.20 ± 0.01 ^b^	30.3 ± 1.0 ^b^	14.8 ± 0.3 ^b^	9.8 ± 0.1 ^b^

a, b in the same column, means indicated by different letters are significantly different (*p* < 0.05).

## Data Availability

The original contributions presented in the study are included in the article. Further inquiries can be directed to the corresponding author.
